# Draft Genome Sequence of a Multi-Metal Resistant Bacterium *Pseudomonas putida* ATH-43 Isolated from Greenwich Island, Antarctica

**DOI:** 10.3389/fmicb.2016.01777

**Published:** 2016-11-08

**Authors:** Fernanda Rodríguez-Rojas, Paz Tapia, Eduardo Castro-Nallar, Agustina Undabarrena, Pablo Muñoz-Díaz, Mauricio Arenas-Salinas, Waldo Díaz-Vásquez, Jorge Valdés, Claudio Vásquez

**Affiliations:** ^1^Laboratorio de Microbiología Molecular, Facultad de Química y Biología, Universidad de Santiago de ChileSantiago, Chile; ^2^Fraunhofer Chile Research FoundationSantiago, Chile; ^3^Facultad de Ciencias Biológicas, Center for Bioinformatics and Integrative Biology, Universidad Andrés BelloSantiago, Chile; ^4^Laboratorio de Microbiología Molecular y Biotecnología Ambiental, Facultad de Química & Centro de Biotecnología Daniel Alkalay Lowitt, Universidad Técnica Federico Santa MaríaValparaíso, Chile; ^5^Facultad de Ingeniería, Centro de Bioinformática y Simulación Molecular, Universidad de TalcaTalca, Chile; ^6^Facultad de Ciencias de la Salud, Escuela de Nutrición y Dietética, Universidad San SebastiánSantiago, Chile

**Keywords:** *Pseudomonas putida*, multi-metal resistance, mercury, tellurite, psychrotroph, Antarctica

## Introduction

At low concentrations, heavy metals and metalloids are highly toxic for most microorganisms (Lemire et al., 2013). Over evolution, bacteria have developed several molecular mechanisms in order to cope with heavy metal/metalloid toxicity (Nies, 2000; Lemire et al., 2013). *Pseudomonas putida* belongs to a group of versatile microorganisms capable to thrive in diverse hostile environments, including multi-metal polluted cold sites (Canovas et al., 2003; Zhang et al., 2012; Moreno and Rojo, 2013). Members of *P. putida* are largely known for their ability to colonize different kinds of environments and to degrade a vast diversity of toxic organic compounds (Wu et al., 2011). In this context, *P. putida* ATH-43 was isolated from soil sediments at the “Prat” Chilean military base located in Greenwich Island, Antarctica, and was recognized as a mercury/tellurite resistant bacterium (Rodríguez-Rojas et al., 2015). Interestingly, this strain shows tellurite resistance only when grown in the presence of mercury, suggesting a cross-resistance mechanism. Further experimental evidence revealed that *P. putida* ATH-43 is highly resistant to other toxicants such as Cd^2+^, Cu^2+^, ${\text{CrO}}_{4}^{2-}$, and ${\text{SeO}}_{3}^{2-}$, and several antibiotics including streptomycin, cefotaxime, kanamycin, and chloramphenicol (Rodríguez-Rojas et al., 2015). On the other hand, global distillation and grasshopper effect are of major worldwide concern since they apparently provide an explanation for the rapid occurrence of heavy metal/metalloids contamination in pristine polar environments (Ebinghaus et al., 2002; Macdonald et al., 2005). In this context, the genome sequence of *P. putida* ATH-43 represents an important information source of genetic resistance determinants to multiple stressors currently affecting the Antarctic ecosystem.

In this report we present the first draft genome sequence of a *P. putida* strain isolated from the Antarctic continent. The shotgun sequencing strategy, assembly, and subsequent annotation showed that the ATH-43 strain possesses a wide spectrum of genetic determinants involved in heavy metal and antibiotic resistance, apparently to cope with extreme oxidative stress conditions. *P. putida* ATH-43 genome now forms part of the 65 genomes of this species registered at the NCBI database (September, 2016) and it is highly related with the endophytic strain *P. putida* W619, which is also resistant to several heavy metals. Further characterization of multi-metal resistant psychrotrophic bacteria such as *P. putida* ATH-43 will be promising to develop novel strategies for heavy metal bioremediation in low temperature environments. All genome data has been submitted to NCBI.

## Materials and methods

### Bacterial isolation and DNA extraction

Bacterium isolation was carried out in LB medium supplemented with increasing concentrations of mercury and tellurite (Rodríguez-Rojas et al., 2015). Briefly, *P. putida* ATH-43 was grown aerobically in LB medium supplemented with 40 μM HgCl_2_ at 25°C for 48 h. DNA extraction was performed using the Wizard® Genomic DNA Purification Kit (Promega). The quality and quantity of genomic DNA was determined by 0.8% agarose gel electrophoresis and by 260/280 nm absorbance ratio using the microplate multireader Tecan Infinite® 200 PRO.

### Phylogenetic tree

Tree was constructed using Maximum Likelihood algorithm by MEGA 6.0 software. Best model was calculated revealing that Jukes Cantor model was the best fit for this nucleotide data set. The option “use all sites” for gaps treatment was also applied. Node numbers represent the per cent of bootstrap replicates of 1000 resamplings (values below 50% are not shown). Sequence alignments was from nucleotide position 24 to 1488 as compared to *E. coli* K12. Scale bar represents 0.01 substitutions per nucleotide positions. Arrow points to the outgroup *E. coli* K12 (accession number AP012306). Accession numbers for all *Pseudomonas* strains included in the study are given in parentheses. Average Nucleotide Identity (ANI) was performed with pyANI using ANIm, which is based on hidden Markov models. For pangenome analysis, GET_HOMOLOGS was used with three clustering algorithms (bidirectional best-hit, COGtriangles, and OrthoMCL). Only congruent results between the three algorithms were used for the final analysis. Strains of *P. putida* and their respective genome accession numbers were W619 (CP000949), SQ1 (JTCJ00000000), F1 (CP000712), KT2440 (AE015451), SF1 (LDPF00000000), BIRD-1 (CP002290), DLL-E4 (CP007620), H8234 (CP005976), and NBRC (AP013070).

### Genome project, sequencing, assembly, and annotation

*P. putida* ATH-43 DNA was submitted to Macrogen® (Seoul, Korea) for next generation whole-genome shotgun sequencing using Illumina Hiseq 2000 platform (January, 2015). Read mapping, *de novo* assembly and genome annotation was performed at the Fraunhofer Chile Research Foundation (Santiago, Chile). DNA sequence was determined by a whole-genome shotgun strategy with a mate pair library of 3 kb (Macrogen®). A total of 10.05 million reads were obtained with an average length of 101 nucleotides. All reads were quality filtered and assembled using the A5 pipeline, an integrated pipeline for *de novo* assembly of microbial genomes (Tritt et al., 2012). The assembled genome of *P. putida* ATH-43 consists of 5.8 Mbp distributed over 260 contigs and organized in 64 scaffolds with fold coverage of 172X.

Open reading frame prediction and annotation was carried out using standard operational procedures (Tanenbaum et al., 2010). Gene models were predicted using Glimmer 3.02 (Salzberg et al., 1998). Predicted coding sequences were annotated by comparison with public databases, BLAST 2.2.31 (Altschul et al., 1990) was used to find homologous sequences with COG, UNIPROT, and NR-NCBI databases, and Hmmer 3.1 was used against PFAM and TIGRFAM. Automatic metabolic reconstruction was carried out using PRIAM software (Claudel-Renard et al., 2003).

## Results

### *P. putida* ATH-43 features

**Figure 1:**
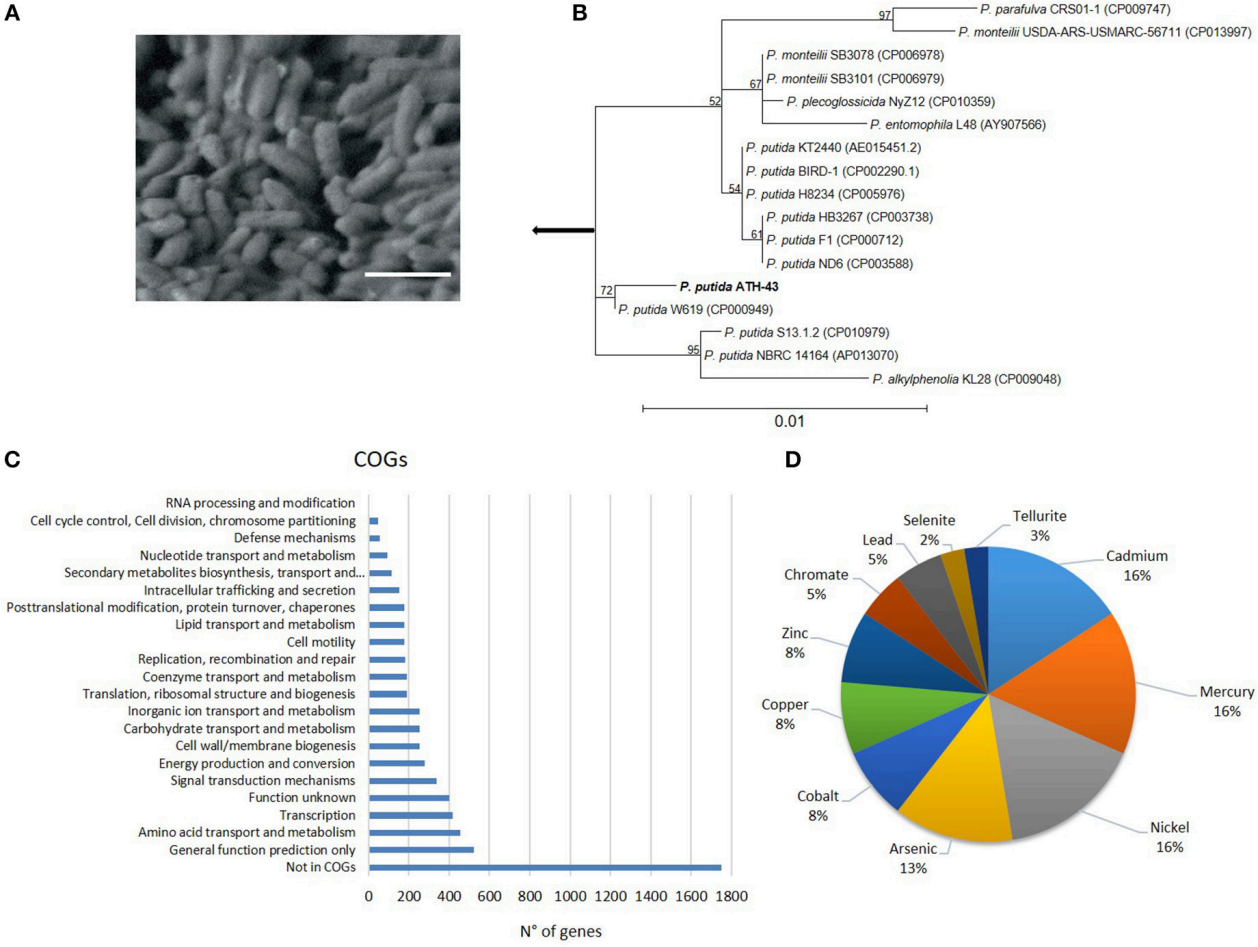
*****P. putida*** ATH-43 morphology, phylogeny, and COG assigned genes**. **(A)** Scanning electron micrograph. Samples were stained with 0.5% (w/v) uranyl acetate and examined using a low-voltage electron microscope (Delong Instruments, LVEM5) with a nominal operating voltage of 5 kV. Bar represents 10 μm. **(B)** Phylogenetic tree, based on the complete 16S rRNA sequence. **(C)** Number of genes assigned to COG categories. **(D)** Pie chart representing the per cent of heavy metal(oid) resistance genes in the *P. putida* ATH-43 genome.

*P. putida* ATH-43 is a Gram negative, non-sporulating, motile, aerobic, rod-shaped (average bacterium dimensions were 6.5 μm length and 2.3 μm width) and psychrotrophic bacterium (Figure 1A) that was isolated from Antarctic sediments at anthropogenic settlements visibly contaminated with oxide compounds. Sequencing of complete 16S rRNA gene revealed phylogenetic affiliation of strain ATH-43 as a member of the *Pseudomonadaceae* family from the *Gammaprotebacteria* class, strongly related to *P. putida* species (Figure 1B). Interestingly, strain ATH-43 forms a distinctive clade with isolate *P. putida* W619, which are clearly differentiated from the rest of *Pseudomonas* isolates, suggesting a different evolutionary ancestral divergence (Figure 1B). In concordance, the Average Nucleotide Identity (ANI) analysis performed with nine selected strains, revealed a close relationship with *P. putida* strains W619 and SQ1 (Supplementary Figure 1A), further contributing to defining the relationship between these bacterial strains. In addition, a pangenome analysis was performed with the same strains that were used for ANI analysis. In this line, ATH-43 strain presented an important number (741) of unique accessory genes, that may contribute to the multi-stress resistance phenotype observed in this strain. Also, a core genome of 1370 genes was shared among the *P. putida* strains, given by their threshold of 90–99% nucleotide identity (Supplementary Figure 1B).

### Whole genome sequence and insights of *P. putida* ATH-43

The assembled genome of *P. putida* ATH-43 consists of 5,830,220 bp, with an average G+C content of 61.5% (Table 1). A set of 79 tRNA genes and two clusters of rRNA genes were identified. From a total of 5124 predicted protein-coding sequences (CDSs), 4436 (86.5%) open reading frames (ORFs) matched coding sequences available in public databases, and 3372 (65.8%) were assigned in clusters of orthologous groups (COG) categories (Figure 1C).

**Table 1:** **Genome properties and features**.

**MIGS ID**	**Property**	**Term**
MIGS-4	Geographical location	Greenwich Island, Antarctica
MIGS-5	Sample collection	January, 2012
MIGS-31	Finishing quality	Draft
	NCBI Bioproject ID	PRJNA278654
	GeneBank ID	LBME00000000
MIGS-28	Library used	Mate-pair of 3 Kb
MIGS-29	Sequencing platform	Illumina HiSeq 2000
MIGS-30	Assemblers	A5
MIGS-32	Gene calling method	Glimmer v3.02
MIGS-31.2	Fold coverage	172X
	Genome size	5,830,220
	G+C content	61.5%
	DNA scaffolds	64
	Total genes	5203
	RNA genes	6
	tRNA genes	79
	Pseudogenes	201
	Protein-coding genes	5124

Open reading frame prediction revealed the presence of multiple genetic determinants related to heavy metals and some metalloids tolerance. This prediction included resistance genes and operons to Hg^2+^, Cu^2+^, Zn^2+^, Ni^2+^, Cd^2+^, Co^2+^, Pb^2+^, ${\text{CrO}}_{4}^{2-}$, ${\text{AsO}}_{4}^{3-}$, ${\text{SeO}}_{3}^{2-}$, and ${\text{TeO}}_{3}^{2-}$ (Figure 1D and Supplementary Table 1), and also genes regarding resistance to antibiotics and drugs such as tetracycline, macrolides, penicillins, aminoglycosides, and streptomycin, among others (Supplementary Table 2). In addition, computational prediction showed the presence of genes encoding seven cold-shock proteins, 773 hypothetical proteins, and an important battery of genes participating in the oxidative stress response, including the unusual mycothiol synthase found exclusively in Gram positive bacteria (Rawat et al., 2007).

The ATH-43 genome harbors more tRNA gene sequences than other known *P. putida* genomes, a trait that may reflect the cell's adaptation to extreme conditions (Wu et al., 2011). In fact, higher tRNA genes content seem to be related with specific cold adaption mechanisms, as determined by comparative genomics analysis (Dutta and Chaudhuri, 2010). In addition, the number of genes involved in signal transduction mechanisms and inorganic ion transport and metabolism (two COG categories) in this bacterium's genome, is similar to other *P. putida* strains but exceeds that displayed by other *Gammaproteobacteria* (Wu et al., 2011). These data may suggest the presence of complex systems of molecular mechanisms controlling gene expression in microorganisms that thrive under highly variable environments.

On the other hand, 13 IS elements were found in the ATH-43 genome sequence using the IS finder tool (www-is.biotoul.fr), along with 21 transposases and 17 integrases (not shown), all elements routinely associated with horizontal gene transfer providing advantage in metal and antibiotic resistance, general stress tolerance, and aromatic compound degradation, among others (Vos et al., 2015; Koonin, 2016). As with tRNA genes, the genome of ATH-43 displays a higher number of genetic determinants involved in metal, antibiotic, and oxidative stress resistance as compared with other reference *P. putida* genomes (Wu et al., 2011), which may be a reflect of the dramatic selective pressure occurring in the Antarctic continent.

The whole-genome shotgun project was deposited in GeneBank and is publicly available since July, 2015 under the accession number LBME00000000 (Direct link: http://www.ncbi.nlm.nih.gov/nuccore/LBME00000000.1).

## Author contributions

FR performed genomic DNA extraction and analyzed the genomic data. PT and JV carried out the *de novo* assembly and gene annotation. EC carried out the ANI and pangenome analysis, and assigned COG categories. PM and MA prepared the samples and took SEM pictures. AU constructed the phylogenetic tree. FR, AU, WD, and CV participated in experiment designing and helped to draft the manuscript. All authors read and approved the final text.

## Funding

FR was funded by the doctoral fellowship “Gastos operacionales” # 21120114 by CONICYT, doctoral support fellowship DG_01-14 by Instituto Antártico Chileno (INACH) and Regular Fondecyt # 1130362. EC was funded by CONICYT+PAI/Concurso Nacional de Apoyo al Retorno de Investigadores/as desde el Extranjero # 82140008, granted by CONICYT, Chile. AU was funded by Ph.D. fellowship and “Gastos Operacionales” # 21120621 granted by CONICYT. MA was funded by Talca funds for Research Initiation (Fondo de Proyectos de Investigación para Investigadores Iniciales).

### Conflict of interest statement

The authors declare that the research was conducted in the absence of any commercial or financial relationships that could be construed as a potential conflict of interest.

## Supplementary material

Supplementary Figure 1 **ANI and pangenome analysis of ***P. putida*** ATH-43. (A)** Heatmap representing the comparison of the average nucleotide identity among ten *P. putida* genomes. **(B)** Pangenome comparison bar chart showing the number of shared genes clustered in cloud (genes in 0–20% of genomes), shell (genes in 20–90% of genomes), soft core (90–99% of genomes), and core genome (100% of genomes).

Supplementary Table 1 **Metal resistance determinants found in the P. putida ATH-43 genome**.

Supplementary Table 2 **Antibiotic resistance determinants found in the P. putida ATH-43 genome**.
