# Supplementary figures and images for: Draft Genome Sequence of a Multi-Metal Resistant Bacterium Pseudomonas putida ATH-43 Isolated from Greenwich Island, Antarctica
Source: Front Microbiol. 2016 Nov 8;7:1777. doi: 10.3389/fmicb.2016.01777 (PMC5099816; doi:10.3389/fmicb.2016.01777)

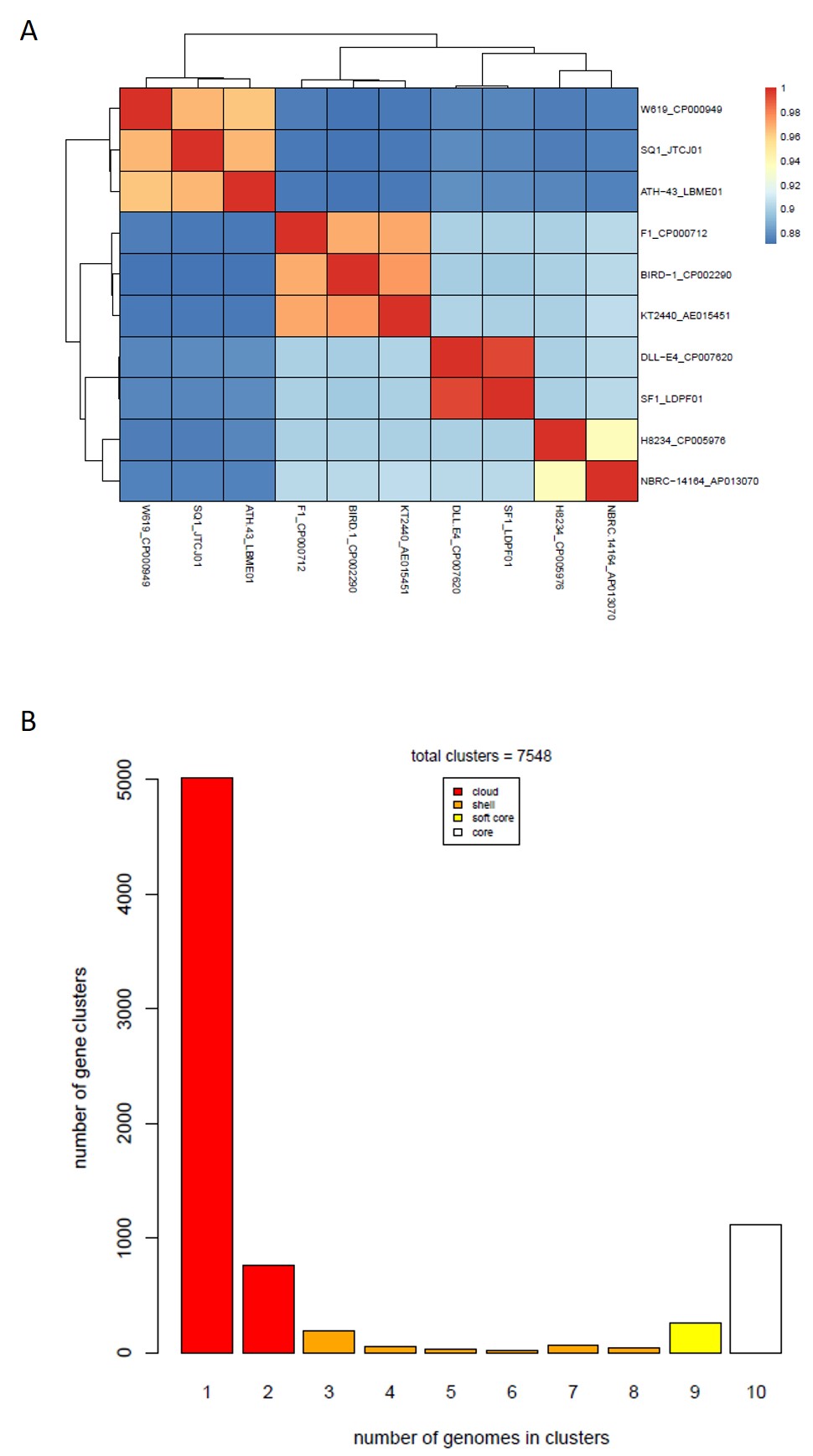

Supplement: Supplementary Figure 1 — ANI and pangenome analysis of P. putida ATH-43. (A) Heatmap representing the comparison of the average nucleotide identity among ten P. putida genomes. (B) Pangenome comparison bar chart showing the number of shared genes clustered in cloud (genes in 0–20% of genomes), shell (genes in 20–90% of genomes), soft core (90–99% of genomes), and core genome (100% of genomes). [file Image1.JPEG]
