# Supplementary material for: Draft Genome Sequence of a Multi-Metal Resistant Bacterium Pseudomonas putida ATH-43 Isolated from Greenwich Island, Antarctica
Source: Front Microbiol. 2016 Nov 8;7:1777. doi: 10.3389/fmicb.2016.01777 (PMC5099816; doi:10.3389/fmicb.2016.01777)
Supplement: Supplementary Table 1 — Metal resistance determinants found in the P. putida ATH-43 genome. [file Table1.DOCX]

Supplementary Table 1. Metal resistance determinants found in the *P. putida* ATH-43 genome

| Protein ID | Predicted function | Size (aa) |
| --- | --- | --- |
| WP_046784398.1 | Copper-resistance protein, CopA family | 461 |
| WP_012316198.1 | Copper-sensing transcriptional repressor CsoR | 105 |
| WP_046784684.1 | Protein ChrB | 313 |
| WP_046784612.1 | Chromate transporter, chromate ion transporter (CHR) family | 449 |
| WP_046784685.1 | Arsenic resistance protein | 366 |
| WP_046785841.1 | Nickel import ATP-binding protein NikE | 273 |
| WP_046785842.1 | Nickel import ATP-binding protein NikD | 256 |
| WP_046785843.1 | Nickel ABC transporter, permease subunit NikC | 281 |
| WP_046785844.1 | Nickel ABC transporter, permease subunit NikB | 313 |
| WP_046785845.1 | Nickel-responsive transcriptional regulator NikR | 138 |
| WP_012314853.1 | Nickel ABC transporter, nickel/metallophore periplasmic binding protein | 483 |
| WP_046785961.1 | Uncharacterized oxidoreductase CzcO-like | 439 |
| WP_046786593.1 | Thiopurine S-methyltransferase, Se/Te detoxification family | 215 |
| WP_046786777.1 | Arsenical resistance operon repressor | 118 |
| WP_046786778.1 | Arsenical resistance protein ArsH | 238 |
| WP_003253425.1 | Cobalt-zinc-cadmium resistance protein CzcC | 394 |
| WP_003253428.1, WP_003139539.1 | Heavy metal efflux pump, CzcA family | 1049-1053 |
| WP_046786982.1 | Cation efflux system protein CzcA | 1017 |
| WP_046787010.1 | Cd(II)/Pb(II)-responsive transcriptional regulator | 147 |
| WP_046787459.1 | Arsenate reductase | 117 |
| WP_017849614.1 | Mercuric resistence transcriptional repressor protein MerD | 97 |
| WP_031633059.1 | Mercuric reductase MerA | 563 |
| WP_003131881.1 | Mercuric resistance protein MerC | 144 |
| WP_003131880.1 | Mercuric transport protein periplasmic component MerP | 92 |
| WP_017849617.1 | Mercuric transport protein MerT | 71 |
| WP_003131870.1 | Hg(II)-responsive transcriptional regulator MerR | 146 |
| WP_010794431.1 | Cd(II)/Pb(II)-responsive transcriptional regulator | 135 |
| WP_024616827.1 | Copper resistance protein B | 349 |
| WP_046787908.1 | Arsenical pump membrane protein | 427 |
