# Supplementary material for: Draft Genome Sequence of a Multi-Metal Resistant Bacterium Pseudomonas putida ATH-43 Isolated from Greenwich Island, Antarctica
Source: Front Microbiol. 2016 Nov 8;7:1777. doi: 10.3389/fmicb.2016.01777 (PMC5099816; doi:10.3389/fmicb.2016.01777)
Supplement: Supplementary Table 2 — Antibiotic resistance determinants found in the P. putida ATH-43 genome. [file Table2.DOCX]

Supplementary Table 2. Antibiotic resistance determinants found in the *P. putida* ATH-43 genome

| Protein ID | Predicted function | Size (aa) |
| --- | --- | --- |
| WP_046784331.1 | Multidrug resistance protein MdtB | 1021 |
| WP_046784313.1, WP_046784333.1, WP_046784346.1, WP_046788222.1, WP_046785657.1, WP_012314115.1 | Bacterial regulatory proteins, TetR family | 169-245 |
| WP_046784686.1 | Drug resistance transporter, Bcr/CflA subfamily | 393 |
| WP_046784877.1 | Multidrug efflux transporter | 154 |
| WP_046784822.1 | Macrolide export ATP-binding/permease protein MacB | 389 |
| WP_046784847.1 | Penicillin-binding protein activator LpoA | 605 |
| WP_046785625.1 | Multiple antibiotic resistance protein MarR | 157 |
| WP_046785450.1 | Beta-lactamase | 379 |
| WP_046784924.1 | Metallo-beta-lactamase superfamily | 285 |
| WP_046786089.1 | Multidrug resistance protein | 399 |
| WP_046785130.1 | Penicillin-binding protein 2 | 629 |
| WP_046784918.1, WP_046784919.1, WP_046784917.1 | Drug resistance MFS transporter, drug:H^+^ antiporter-2 (DHA2) family | 457-525 |
| WP_046787854.1 | Penicillin-binding protein 1C | 766 |
| WP_046788034.1, WP_046788086.1 | Multidrug DMT transporter permease | 285-293 |
| WP_046788176.1 | Penicillin-binding protein 1B | 748 |
| WP_046786991.1 | Penicillin amidase | 767 |
| WP_046788159.1 | Glyoxalase/bleomycin resistance protein/dioxygenase superfamily | 137 |
| WP_046784587.1 | Fosmidomycin resistance protein | 403 |
| WP_001082319.1 | Streptomycin 3''-kinase | 267 |
| WP_046785721.1, WP_052750419.1 | Aminoglycoside phosphotransferase | 395 |
| WP_000480968.1 | Aminoglycoside/hydroxyurea antibiotic resistance kinase | 120 |
